# Uncrewed aerial vehicle with onboard winch system for rapid, cost-effective, and safe oceanographic profiling in hazardous and inaccessible areas

**DOI:** 10.1016/j.ohx.2024.e00518

**Published:** 2024-03-16

**Authors:** Ebbe Poulsen, Søren Rysgaard, Karina Hansen, Nanna B. Karlsson

**Affiliations:** a Arctic Research Centre, Department of Biology, Aarhus University, Ole Worms Allé 1, DK-8000 Aarhus C, Denmark; b Geological Survey of Denmark and Greenland, Øster Voldgade 10, DK-1350 Copenhagen K, Denmark

**Keywords:** Uncrewed Aerial Vehicle, Aerial Winch System, Ocean profiling, Hazardous areas, Remote operation, Marine-terminating glaciers

## Abstract

Interactions between coastal waters and marine-terminating glaciers in the Polar Regions play a significant role in global sea level rise fueled by a rapidly warming Arctic.

The risk of glacier calving, and the abundance of ice, can make it impossible for surface vessels to access the waters near glacier termini. Alternative methods using manned aircraft are expensive. As a result, oceanographic measurements are limited near glacier termini.

We present an uncrewed aerial vehicle (UAV) with an on-board winch system that allows oceanographic profiling in remote, hazardous areas using a commercial conductivity, temperature, and depth (CTD) sensor payload. The UAV is optimized for easy handling and deployment and is capable of high-speed and efficient cruise flight. An autopilot system provides pilot assistance and autonomous flight capabilities. The total weight of the UAV including payload is 6.5 kg with an endurance of 24 min.

Testing of the system was conducted in South Greenland during winter conditions in March 2023 with successful profiles collected near a glacier terminus (<5 m) and in small openings in ice mélange (2.2 m). The system proved capable, reliable, and efficient. Further development of the system will allow other sensors for an even more flexible measurement suite.

## Graphical abstract

**Figure:**
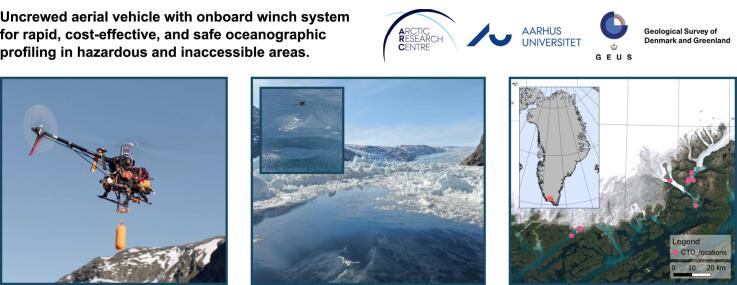

## Specifications table

**Table:** 

Hardware name	Arctic Research Centre Aerial Winch System (ARC-AWS)
Subject area	Environmental, planetary and agricultural sciences
Hardware type	Field measurements and sensors
Closest commercial analog	Velos V3 with Daiwa Winch
Open source license	CC BY 4.0
Cost of hardware	€ 5,700
Source file repository	https://doi.org/10.17632/j2z3ns9hn4.2

## Hardware in context

Accelerated melting of the Polar ice sheets due to climate change has caused a need for further understanding of how the increased freshwater input from marine-terminating glaciers affects the ocean locally and globally [1]. Currently, there is a lack of knowledge of how, where and when meltwater is released from glaciers into the fjords, and whether warm ocean water from the coast reaches the glacier front [2]. The main reason for this knowledge gap is a scarcity of measurements close to glacier termini due to their general inaccessibility and the risk of glacier calving [3]. Furthermore, seasonal variations are not well understood due to a dearth of measurements in the winter period [4], [5].

Oceanographic profiling is commonly carried out using crewed marine vessels with winches on board that lower the conductivity, temperature, and depth (CTD) instruments through the water column. These vessels cannot go near glacier termini due to the difficulties of maneuvering in the ice mélange as well as the danger of glacier calving. Uncrewed surface vehicles (USV) such as the Jetyak have been used as a successful alternative [6], [7] but are limited to waters without significant ice mélange as often seen during winter.

Autonomous underwater vehicles (AUV) can reach locations of interest with various payload options, but equipment costs upwards of €500,000 limits the use in high-risk areas. AUVs that cost less than €50,000 are increasing in numbers [8], [9], [10] and pose a viable option. However, their lower speed limits operational efficiency and the need for access to water for deployment limits the possible operational locations close to glacier termini.

Measurements have been collected from crewed aircraft using expendable CTD’s (XCTD) from Sippican, Inc. [3], [7], [11]. The XCTD profiler is a single-use device that descends at a known velocity while transmitting temperature and conductivity measurements to a receiver through a wire. The wire breaks when fully extended, thus discarding the instrument. The high cost of equipment replacement and aircraft hire is a limiting factor to this method.

Attempts have been made to modify the XCTD concept to address these shortcomings, resulting in the ARC-TOP concept [12]. This is a low-cost reuseable CTD for UAV deployment and retrieval that uses a single-shot buoyancy engine in place of a winch system. The ARC-TOP is free-flowing and therefore susceptible to currents displacing it laterally during profiling. In areas of high currents or significant ice coverage this introduces a considerable risk of instrument loss. Further, handling the instrument between deployments was found to be time-consuming [12].

The use of a UAV to both transport the instrument to the correct measurement location and as the profiling engine seems to be a relevant concept to pursue that can alleviate the issues with other methods. The resulting UAV requirements are listed in Table 1. Commercial solutions such as the Velos V3 UAV [13] with a Daiwa winch [14] could be modified to fit the mission profile, but the cost (> €90,000) and the size of the UAV are outside the requirements. Open-source options with similar mission profiles exists [15] but fail to meet several requirements (dimensions, battery capacity, profiling depth).

**Table 1:** Main ARC-AWS requirements and specifications.

**Requirement**	**Value/specification**	**ARC-AWS**	**Remark**
Dimensions (shipping)	Max. 1,400x450x250 mm	1,400x450x250 mm	Largest transport case allowed. Should fit without major disassembly
Deployment location	From land	From land	
Deployment time	Max. 30 min	≈ 20 min	Time from unpacking to takeoff
Handling	2 pers.	2 pers.	Pilot and spotter
Temperature	−10 °C to 25 °C	−6 °C to 20 °C	Only tested in specified interval
Wind	8 m/s	7 m/s	Only tested to specified value
Flight time	Min. 20 min	24 min	Recorded in stationary hover. Flight time is increased with cruise flight
Battery capacity	Max. 450 Wh	311 Wh	Limited to simplify operations
Operational range	Min. 3 km	6 km	Max. possible distance from takeoff to measurement location
Cruise speed	Min. 15 m/s	16 m/s	
Takeoff weight		6.5 kg	No requirement imposed
Payload capacity	Min. 0.5 kg	0.5 kg	Payload can be increased if shorter flight time is acceptable
Profiling depth	Min. 100 m	100 m	
Camera	Realtime 720p video feed	Realtime HD video feed	
Price	Max. €15,000	€5,700	Excluding sensor payload

## Hardware description

The Arctic Research Centre Aerial Winch System (ARC-AWS) described here is a compact, low-cost, versatile, and easy-to-use UAV platform with an on-board autonomous winch. The winch is connected to a SonTek CastAway CTD sensor [16] but any payload that is within the spatial constraints is applicable.

The typical mission profile consists of a takeoff and outbound cruise of up to 6 km, followed by a hover 2–5 m above the measurement location. Once the hover is established, the CTD sensor is lowered towards the water surface by the winch. When the sensor is at the surface of the water, the winch motor disengages, and the sensor descends at terminal velocity through the water column while extending the winch line. The UAV remains in a stationary hover during profiling. At a set depth, or on pilot input, the winch motor engages and reels in the sensor. Once profiling is complete, the pilot commands the UAV to carry out the inbound cruise and subsequent landing. Data can be downloaded from the instrument after disarming the UAV. The UAV can be flown manually using the onboard camera for navigation or autonomously using user-defined waypoints.

**Fig. 1:**
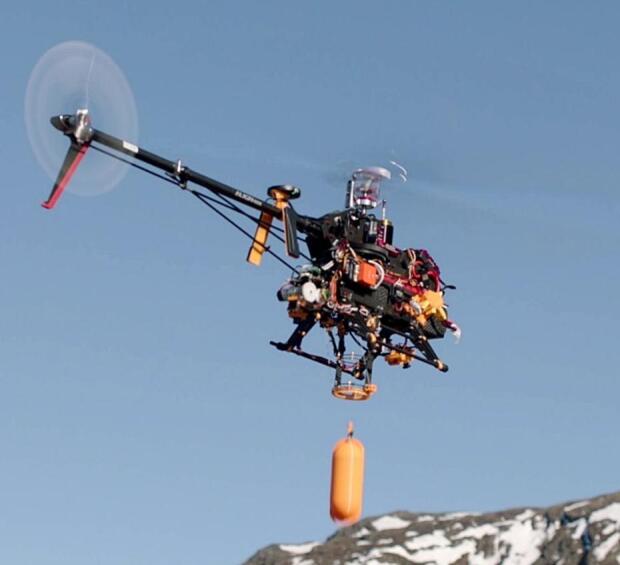
The complete ARC-AWS UAV with CTD payload (orange) during a test flight. Photo curtesy of Lars Ostenfeld. (For interpretation of the references to colour in this figure legend, the reader is referred to the web version of this article.)

**Fig. 2:**
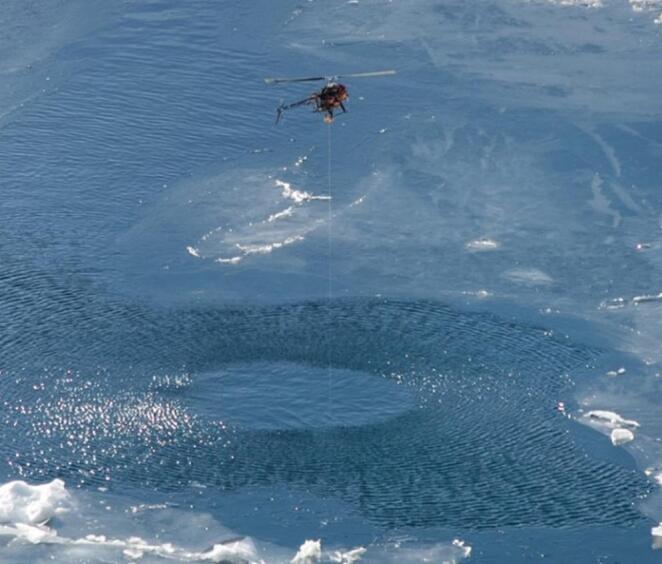
Profiling operation in areas with significant ice coverage. Note the wire extending from the UAV to the submerged CTD sensor.

Specifications for the ARC-AWS can be found in Table 1. The complete UAV with payload and a profiling operation can be seen in Fig. 1 and Fig. 2, respectively.

Key advantages of the ARC-AWS concept are:

• Cost-efficient access to oceanographic profiling in highly hazardous and inaccessible areas.
  o Waters with significant ice mélange.
  o Waters in close proximity to glacier termini.
• Rapid deployment allowing for optimized field campaigns.
• Very compact and lightweight setup for easy transport in small vehicles.
• Efficient cruise flight enabling long-range flights and allowing for greater measurement radius from a single operating point.

The ARC-AWS consists of three main parts: UAV platform, winch system, and payload. Each will be presented in detail in the following.

### UAV

The UAV platform consists of a modified kit helicopter, the Align T-Rex 650X [17]. This aircraft is designed for aerobatic remote-controlled flight but was found to be a suitable fit for the needed use-case. The original aircraft use-case ensures all components are designed to withstand significantly higher loads than expected for the mission profile.

**Fig. 3:**
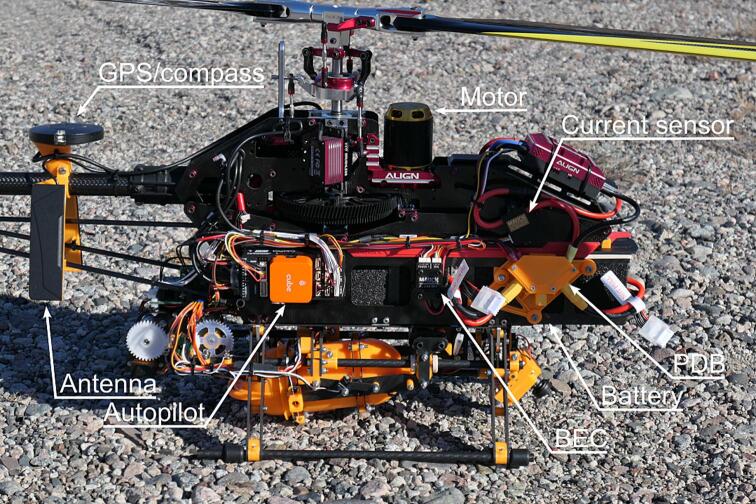
UAV with additional and modified components annotated. The radio system is mounted to the opposite side of the frame.

**Fig. 4:**
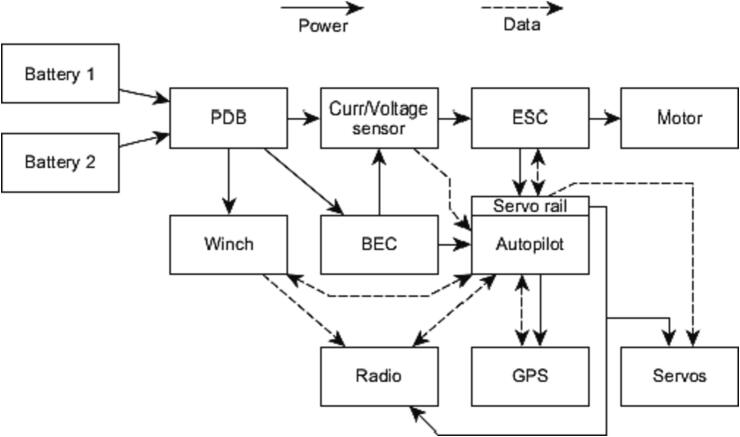
UAV system architecture.

Additional instrumentation was added to the UAV for increased operational reliability, as shown in Fig. 3. A Cube Orange autopilot system [18] running open source ArduPilot software [19] was added to enable autonomous flight and pilot assistance. This system uses a GNSS receiver and compass [20] along with a barometer, accelerometer, and gyroscope integrated in the autopilot for positional awareness. A current and voltage senor [21] provides battery monitoring to the autopilot. The entire system is powered by two 22.2 V, 7 Ah, lithium-polymer batteries [22] connected in parallel via the power distribution board (PDB). The electrical system is outlined in Fig. 4. Radio control, telemetry and video transmission is done by the Herelink system [23].

Some modifications have been made to the original aircraft to optimize it for the mission profile. The stock motor is significantly oversized for the intended mission, thus it was replaced by a lighter version that is designed for lower RPM [24], thereby increasing efficiency [25], [26]. To reduce weight further, the canopy and associated hardware was removed. The stock landing skids were removed as the custom payload incorporates landing skids in the design (see section 2.2). Two isolated battery boxes were added to allow the pre-heated batteries (see section 6) to remain at an optimum working temperature during flight.

The autopilot continuously logs a comprehensive list of parameters relating to the UAV performance, health, and status. Additionally, the winch controller relays information about its status to the autopilot for logging and transmission to the pilot. A custom Lua script handles this additional information.

Selecting a conventional helicopter configuration in favor of the more commonly used multi- or quadcopter configuration is based on efficiency, mission profile, operating environment, and logistics. The efficiency of a rotorcraft generally increases with lower disc loading, as is the case for helicopters compared to multi- or quadcopters [27]. Further, increased cruise efficiency and speed make the helicopter more ideally suited for missions profiles with significant cruise segments and medium to high weight payloads [25]. Wind and turbulence stability are important for operation in the frequently harsh environment of the Polar regions, requiring rapid response to control inputs. The control system inertia for a UAV in the relevant size range is higher in multi- or quadcopters as rotational speed of the rotors must be changed. For helicopters, only the pitch of the rotor blades is changed, resulting in a significant increase in control response speed [28]. Finally, transport to the operating locations is often carried out in small boats or aircraft with very limited cargo space. This favors a long, narrow package that can more easily be stowed in various positions. The helicopter configuration natively fits this requirement. Disadvantages include an increased mechanical and operational complexity potentially increasing the risk of UAV failure. Furthermore, development of payloads and autopilot tuning is more time-consuming for helicopters. For our purposes the advantages of the helicopter configuration outweighed the disadvantages, which is why this configuration was selected.

### Winch

**Fig. 5:**
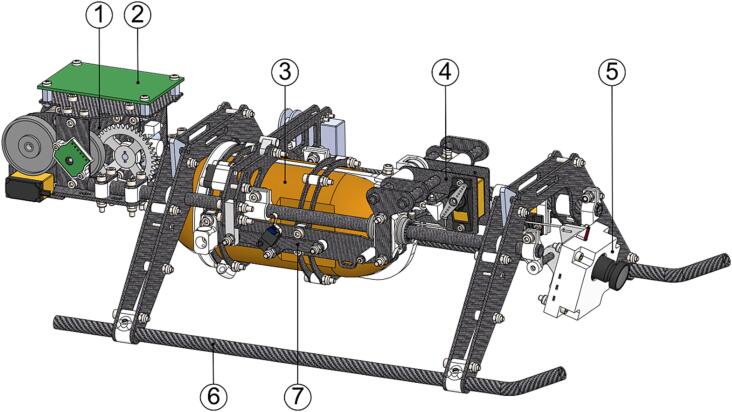
Winch assembly consisting of (1) winch, (2) winch controller PCB, (3) sensor payload, (4) sensor catch mechanism, (5) gimbal and camera assembly, (6) landing skid, and (7) sensor pivot mechanism.

The complete winch assembly serves as the sensor deployment mechanism, camera and gimbal, and landing skids. The winch and payload are detachable from the main UAV frame using a set of spring-loaded quick release hooks for easy storage and logistics. The isolated winch assembly with a CTD sensor payload is seen in Fig. 5. The winch system uses a 0.28 mm fishing line with a stated breaking tension of 7 kg. This enables a small spool, reducing size and weight. Further, the assembly structure is composed of mainly carbon fiber composite tube and plates for a strong, light-weight frame.

**Fig. 6:**
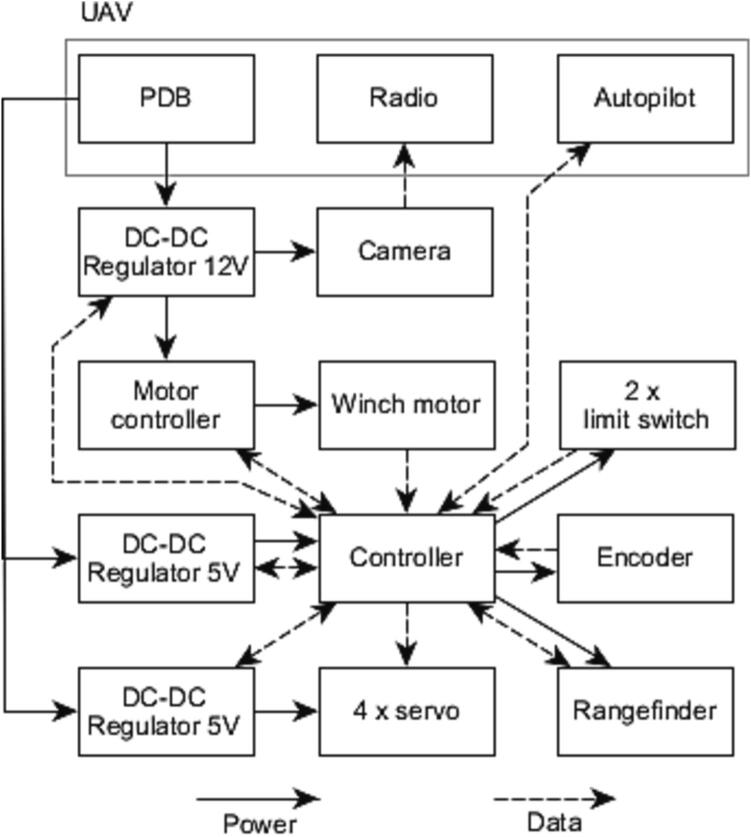
Winch electrical system architecture.

An overview of the electrical system of the winch assembly can be found in Fig. 6.

#### Pivot mechanism

**Fig. 7:**
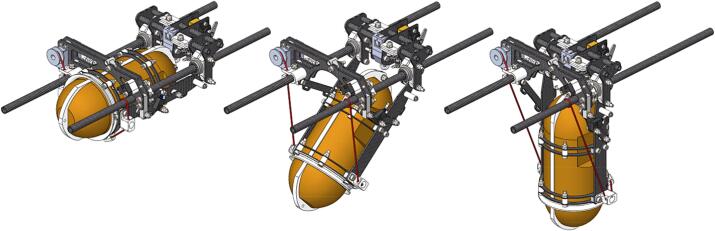
Sensor transition from horizontal (left) to vertical (right). The transition is controlled by a servo with a cord (red) attached to the Scott Russel linkage. (For interpretation of the references to colour in this figure legend, the reader is referred to the web version of this article.)

Central to the winch assembly is the pivot mechanism that transitions the sensor from horizontal during takeoff, cruise, and landing to vertical as needed for profiling. It was found during testing that vibration and flexibility in the sensor attachment severely limited the maximum UAV cruise speed by introducing instability and increasing drag. Using a horizontal sensor position during high-speed flight segments enables the sensor center of mass to remain as close as possible to the UAV main rotor, improving maneuverability, while also clamping the sensor in a secure, rigid position that minimizes vibration and flexibility. A Scott Russel linkage is used to keep the sensor center of mass at the same horizontal position during transition. A sensor transition is shown in Fig. 7. A servo controls the transition through a cord connected to the mechanism. Limit switches are used at both ends of travel to detect full movement.

The pivot mechanism is mounted to the landing skid via two carbon fiber tubes that also function as linear guides for the pivot mechanism and a mounting point for the winch unit and camera gimbal.

A catch mechanism is integrated within the pivot mechanism and is used to secure the sensor during the sensor transition. It consists of a spring-loaded latch that can be opened by the incorporated servo and a hook connected to the sensor payload. During transition or when the sensor is horizontal the latch is closed. When the sensor is vertical and ready for deployment tension is applied to the winch line and the latch is opened, allowing the sensor to reel out. When deployment is complete, and the sensor is reeled in, the hook engages with the latch. Incorporated within the catch mechanism is a knife that can be used to release the sensor payload from its connection to the UAV. This is a mitigation against emergencies where there is a risk of losing the UAV.

#### Winch unit

**Fig. 8:**
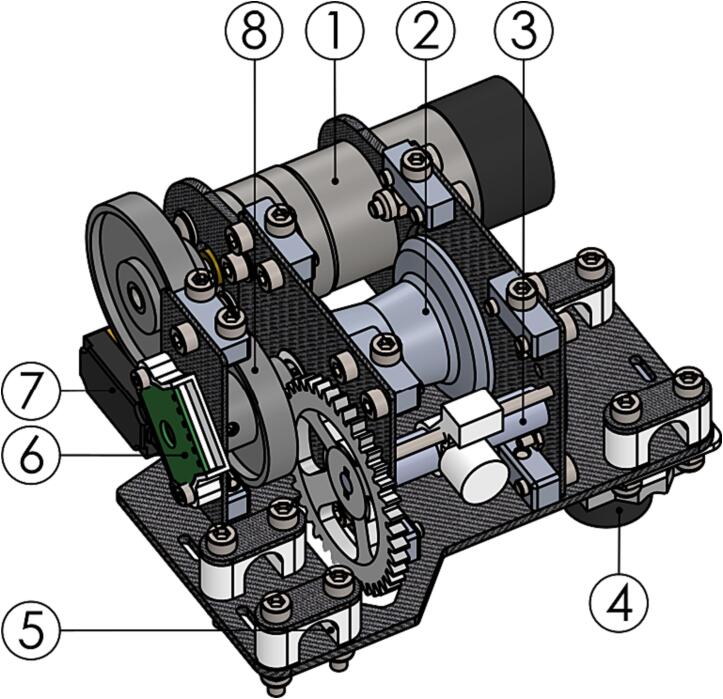
Winch unit with top plate and controller PCB hidden. (1) Motor, (2) spool, (3) level wind, (4) rangefinder, (5) mounting clamp, (6) spool encoder, (7) clutch servo and (8) gearbox.

The winch unit is based on the spool and level wind assembly from a commercial fishing reel [29]. A DC gearmotor is connected to the spool through a single stage gearbox. The motor mount houses a simple clutch that can disengage the motor gear from the spool gear to allow the payload to fall at terminal velocity. Two rotary encoders are connected to both motor and spool shaft for position feedback. The winch assembly also houses a downward facing rangefinder and the winch controller PCB. See Fig. 8 for an overview of the winch unit.

#### Gimbal and camera

A light-weight camera is mounted on a 1-axis gimbal to supply the pilot with a video feed from the UAV. A scroll wheel on the UAV controller allows control of the gimbal servo to angle the camera vertically. This allows the pilot to see forward for cruise and downwards for deployment.

#### Controller

The complete winch assembly is controlled by the winch controller. At the core of this is a Teensy 4.0 development board [30] that manages communications with the autopilot and controls the winch motor, servos, and sensors. Software for the Teensy 4.0 is written in the C++ language using the Arduino framework. After initialization the controller runs several processes at specified frequencies, including a finite state machine. A description of the main processes is included in Table 2**.** A further description of the state machine states is listed in Table 3.

**Table 2:** Main processes for the winch controller.

**Process**	**Frequency [Hz]**	**Function**
State machine	20	Main system control and monitoring based on sensors and user input.
Outer winch control loop	100	PID controller for winch motor speed. The output is a motor current that is passed to the inner winch control loop.
Inner winch control loop	4000	PID controller for winch motor current. The output is a PWM value that is used to directly control the winch motor.
MAVLink receive	100	Check for incoming data from the autopilot. Parse and handle any available data or commands.
MAVLink send telemetry	5	Send winch system information to the autopilot for logging. Information include state, line length and winch control loop status.
Rangefinder	10	Read rangefinder measurement and relay the data to the autopilot.

**Table 3:** States of the controller state machine.

**State**	**Description**	**Entered if**
IDLE	Await commands from user while the sensor is parked and secure	Sensor is parked
PIVOT	Move the sensor to either parked (horizontal) or extended (vertical). Automatically switch state upon completion.	User commands reel in/out and state is IDLE or CATCH
CATCH	Control the catch mechanism. Automatically check whether the sensor is properly secured before switching to PIVOT state if reeling in.	User commands reel in/out and state is PIVOT or REEL
REEL	Control winch motor speed based on line length and user input. Automatically detects sensor reel in complete.	User commands reel in/out and state is CATCH or DIVE
DIVE	Disengages clutch to allow sensor to fall at terminal velocity.	User commands dive and state is REEL
CUT	Cuts sensor line to prevent loss of UAV in emergency situations.	User commands cut
ERROR	Stop winch motor and broadcast error information.	Error detected by the system

### Payload

The UAV and winch assembly have been designed specifically for the SonTek CastAway CTD sensor. This is a compact and rugged CTD instrument with outside dimensions of Ø65x152.5 mm and a weight of 0.53 kg. However, any payload that is within these constraints can be fitted to the ARC-AWS with little to no modifications as the payload is a self-contained unit. A prototype water sampler has been used successfully with the ARC-AWS as well.

Instrument weight is currently limited by the winch motor torque. If a fixed payload, e.g., scanner or atmospheric sensors is used the maximum weight increases as the UAV is capable of significantly higher loads at the cost of flight duration.

### Price

**Fig. 9:**
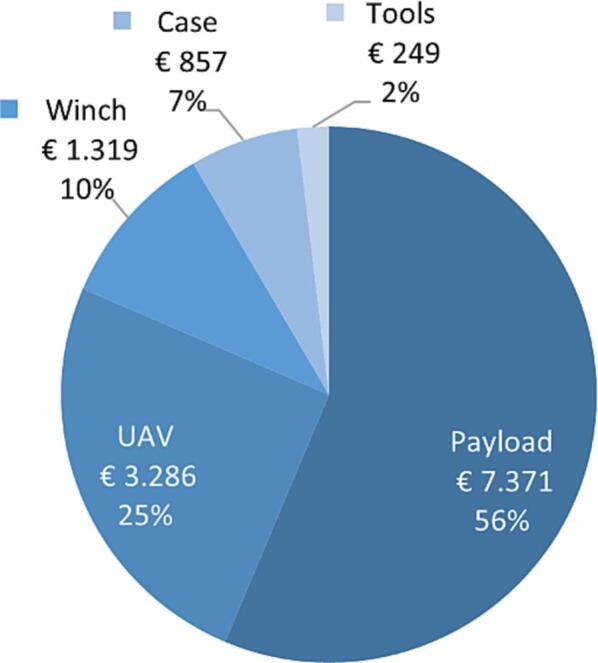
Cost breakdown of the ARC-AWS excluding VAT based on data from the BOM (see design file Naming_and_BOM.xlsx).

Fig. 9 provides a breakdown of the total cost of the ARC-AWS. As seen, the sensor payload is by far the most significant factor in determining the total cost. The UAV with winch assembly and accessories amounts to € 5711, excluding cost of development and assembly. Including a SonTek CastAway CTD the total cost is € 13082.

## Design files summary

All files for construction and development are located at an online repository (https://doi.org/10.17632/j2z3ns9hn4.2). The repository is divided into various sub-folders each with a read-me file with information regarding the specific design files in that directory. See Table 4 for a summary of the main design files.

**Table 4:** Summary of included design files for the ARC-AWS. The listed files are intended as a point of entry into the remaining design files.

**Design file name**	**File type**	**Open source license**	**Location of the file**
Naming_and_BOM.xlsx	Excel Workbook	CC BY 4.0	https://doi.org/10.17632/j2z3ns9hn4.2
AWS-300-UAV.sldasm	CAD file	CC BY 4.0	https://doi.org/10.17632/j2z3ns9hn4.2
AWS-230-Winch.kicad_pro	KiCad Project	CC BY 4.0	https://doi.org/10.17632/j2z3ns9hn4.2
AWS-240-Winch_controller.sln	Visual Studio solution	CC BY 4.0	https://doi.org/10.17632/j2z3ns9hn4.2
AWS-240-Winch_controller.ino	Arduino Sketch	CC BY 4.0	https://doi.org/10.17632/j2z3ns9hn4.2
AWS-320-Autopilot_parameters.param	ArduPilot parameter file	CC BY 4.0	https://doi.org/10.17632/j2z3ns9hn4.2

*Naming_and_BOM.xlsx* contains a complete list of design files and a complete BOM. Supplementary information including naming convention for design files and price calculations is also available here.

*AWS-300-UAV.sldasm* contains a 3D model of the entire winch and relevant parts of the UAV. A STEP file is also located at the same location.

*AWS-230-Winch.kicad_pro* is a KiCad project with documentation for PCB manufacturing for the winch controller.

*AWS-240-Winch_controller.sln* is a solution file for the code editor Visual Studio Community [31] with the Visual Micro plugin [32] used for development of the firmware onboard the winch. This links all used libraries to the main Arduino Sketch file, *AWS-240-Winch_controller.ino*.

A list of all parameters used by the UAV autopilot is included in *AWS-320-Autopilot_parameters.param.* Note that these are provided as a reference for peripheral setup only. Control tuning is needed even for direct copies of the ARC-AWS. Failure to properly tune the UAV can lead to severe damage or injury.

## Bill of materials summary

Table 5 contains a summary of the main components used in the ARC-AWS. A full-length BOM is found at the linked repository in the file *Naming_and_BOM.xlsx*.

**Table 5:** Summary of the complete BOM for ARC-AWS only containing the 15 most expensive entries. For a complete version see Naming_and_BOM.xlsx in the supplied repository.

**Designator**	**Component**	**Number**	**Cost per unit - EUR**	**Total cost - EUR**	**Source of materials**	**Material type**
P84	AWS-510-CastAway	1	7.370,88	7.370,88	https://mjk.com/da	Other
P1	T-REX 650X SUPER COMBO − 12S	1	975,64	975,64	https://rotordisc.dk/shop/t-rex-650x-super-11076p.html	Composite
P5	Herelink - HD Video Transmission System V1.1	1	866,15	866,15	https://www.3dxr.co.uk/autopilots-c2/the-cube-aka-pixhawk-2-1-c9/herelink-hd-video-transmission-c16/cubepilot-herelink-hd-video-transmission-system-v1-1-p5226	Semiconductor
P224	Zarges K470 40,848	1	801,42	801,42	https://flightcases.dk/cases/other-cases/zarges-k470-40848.html	Metal
P4	Cube Orange + Standard Set with ADS-B Carrier Board	1	336,84	336,84	https://www.3dxr.co.uk/autopilots-c2/the-cube-aka-pixhawk-2-1-c9/cube-autopilot-and-combos-c10/cubepilot-the-cube-orange-standard-set-with-ads-b-carrier-board-p5328	Semiconductor
P85	Tattu Lipo 6S 7000mAh 22.2 V 25C Battery pack with XT90	2	121,62	243,24	https://www.gensace.de/tattu-lipo-6s-7000mah-22-2v-25c-battery-pack-with-xt90.html	Polymer
P64	Herelink Industrial Drone 4 K Split camera with HDMI output, EIS 2.0	1	184,77	184,77	https://fireflycameras.com/products/industrial-drone-4k-camera-with-hdmi-output-and-eis-2-0	Semiconductor
P8	Here 3 Special - with 60 cm CAN cable	1	144,36	144,36	https://www.3dxr.co.uk/	Semiconductor
P54	Penn Squall Low Profile	1	139,27	139,27	https://www.topgrej.dk/penn-squall-low-profile.html	Metal
P3	Scorpion HK-3536-510KV Motor	1	120,69	120,69	https://www.live-hobby.de/en/1-sorto/Scorpion-HK-3536-510KV-Brushless-Motor-SP-HK3536-0510.html	Metal
P36	AWS-310-Battery_box_panel-Top	2	43,44	86,88	https://tech-part.com/	Composite
P31	AWS-310-Battery_box_panel	2	33,69	67,38	https://tech-part.com/	Composite
P132	AWS-210-Spool_bushing	1	67,01	67,01	Workshop	Metal
P194	Lithium Battery Charger	1	65,10	65,10	https://bluerobotics.com/store/comm-control-power/powersupplies-batteries/lithium-battery-charger/	Metal
P53	Reefs RC 99Micro Servo Winch w/Micro Spool Kit	1	65,09	65,09	https://reefsrc.com/products/99micro-servo-winch	Semiconductor

Parts made in-house (mainly 3D-printed or minor material modifications) have a listed price of € 0. The cost of the material is listed in the BOM separately. The source of materials for such parts refers to the designator of the material entry for reference.

## Build instructions

It is recommended to use the included assembly CAD file for reference during assembly. Short videos documenting the build procedure are also included for selected assemblies in the *Build_Instructions* directory. The UAV and winch can be assembled separately. General considerations throughout the assembly include:

- Cutting and shaping of carbon fiber parts is required during assembly. Use protective equipment when necessary.
- Assembling the battery container requires the use of epoxy adhesive. Use appropriate protective equipment as stated in the safety data sheet for the specific adhesive.
- Soldering of electronics should be carried out in a well-ventilated area.
- The winch wire is strong and durable but is susceptible to failure if scratched. Take care during assembly and handling to protect the wire to prevent accidental sensor loss.
- 3D-printed parts have been printed on consumer grade FDM printers. All 3D-printed parts must be checked for defects post printing and support material must be removed if used.
- Thoroughly clean any surface that must bond with adhesive tape or epoxy with isopropyl alcohol prior to bonding. Use in a well-ventilated area.

Technical drawings of machined parts are included in the supplied repository. The assembly is completed using the fasteners specified in Table 6.

**Table 6:** Fasteners used in the ARC-AWS.

**Standard**	**Sizes**	**Description**
DIN 471	16X1	Retaining ring
DIN 7982	2.2x6.5	Countersunk self-tapping screw
ISO 10,673	2.25, 3.2	Plain washer
ISO 4029	3X5	Set screw
ISO 4035	M2, M3	Nut
ISO 4762	2X6 to 2X20	Cylindrical socket head screw
ISO 4762	3X8 to 3X20	Cylindrical socket head screw
ISO 7040	M3	Locking nut
ISO 7380	3X6, 3X12	Button socket head screw
ISO 8734	3X16	Parallel pin

### UAV

Follow the instructions below to assemble the UAV with added instrumentation. Please review the ArduPilot documentation prior to and during assembly [33].

1. Assemble the Align T-Rex 650X helicopter (P1) per the instructions included with the kit, only deviating at the following points:
  a. Page 9: *Canopy Mounting Bolt* can be omitted if no canopy is used.
  b. Page 9: Extend sanding recommendation to edges of all carbon fiber frame parts to prevent wire damage.
  c. Page 10: *Canopy support* and *Canopy Spacer* can be omitted if no canopy is used.
  d. Page 10: Insert 2 mm spacers between the six *Frame Mounting Blocks* and the *Bottom Plate*.
  e. Page 10: *Canopy Double Sided Tape* can be omitted if no canopy is used.
  f. Page 11: Install two *AWS-310-Payload_mount* (P2) instead of the indicated *Landing Skid* and associated hardware. Insert two 3x16 parallel pins in *AWS-310-Payload_mount*.
  g. Page 13: Install the custom motor (P3) instead of the one included with the kit. Four new holes must be drilled in the *Motor Mount* to accept the custom motor.
  h. Page 19: Do not install the *Microbeast PLUS Flybarless System* as this is redundant when using an autopilot.
  i. Page 20: Do not install the *Battery Mount* and do not apply hook and loop tape to the batteries.
  j. Page 20: Do not install the canopy if not intended.
2. Install the Cube Orange autopilot (P4) as shown in Fig. 3 using the included double-sided foam tape.
3. Install the Herelink Air Unit (P5) roughly opposite the Cube Orange using double sided tape (P6) with the antenna connectors pointed towards the tail rotor.
4. Mount the Herelink Air Unit antennas on *AWS-310-Antenna_mount* (P7) using double sided tape (P6) and fasten the Here 3 GPS unit (P8) on *AWS-310-Antenna_mount_top* (P9) using the screw on the bottom of the GPS unit. Combine the two assemblies around the tail boom and clamp with a M3 bolt and locking nut. Use zip ties (P10) to further fix the assembly to the tail support rods and connect the GPS and antenna cables to the autopilot and Herelink Air Unit respectively. See Fig. 3 for reference.

To assemble the power distribution board and associated power components, do the following:

1. Solder three male XT90 connectors (P11) to the two *AWS-335-PDB_rail* (P12) on the outside face so that the connectors fan out. Make sure to align the positive and negative connector terminals.
2. Insert the assembly into *AWS-310-PDB_front* (P13) as shown in Fig. 10 and secure with fasteners.

**Fig. 10:**
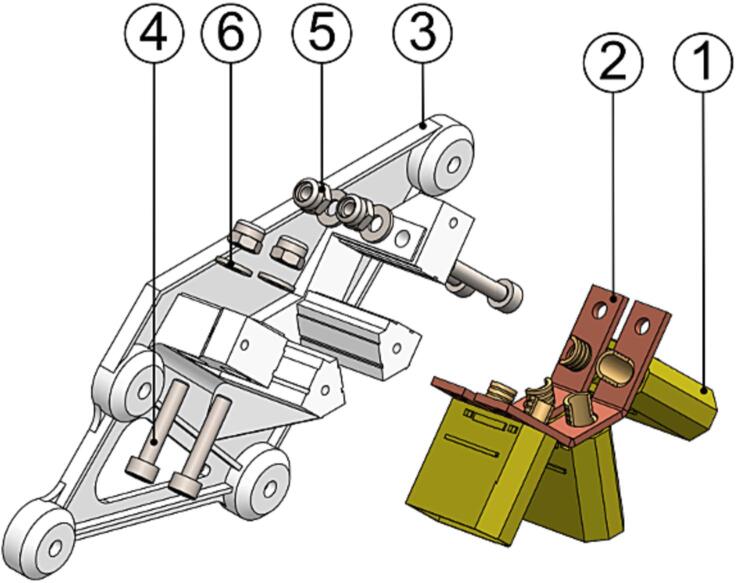
PDB assembly step 2 exploded view with (1) XT90 connector, (2) AWS-335-PDB_rail, (3) AWS-310-PDB_front, (4) M3x16 bolt, (5) M3 locking nut and (6) washer marked.
3. Clamp the assembly to the front spoke of the UAV lower right frame by using *AWS-310-PDB_backplate* (P14). Cover the exposed power rails by installing *AWS-310-PDB_cover* (P15). See Fig. 11 for reference.

**Fig. 11:**
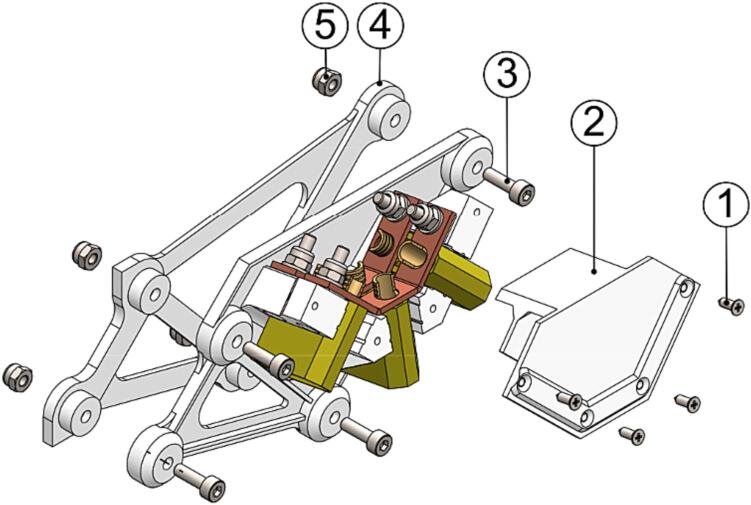
PDB assembly step 3 exploded view with (1) 2.2x6.5 screw, (2) AWS-310-PDB_cover, (3) M3x10 bolt, (4) AWS-310-PDB_backplate and (5) M3 locking nut marked.

Installing power management components and wiring is done as described:

1. Solder the MAUCH current sensor (P16) to the positive (red) ESC cable. Install a cable lug (P17) on the PDB side of the current sensor and on the ESC negative lead (black).
2. Mount the current sensor and MAUCH BEC (P18) to the right-side UAV frame using double sided tape (P6). Use Fig. 3 as a reference for mounting location. Use the cable included with the current sensor to connect to the BEC.
3. Assemble a 350 mm power cable for the winch controller by cutting and soldering pre-crimped Molex Nano-Fit cable (P19) to 16AWG cable (P20). Inset the crimped leads into a 2-circuit receptacle housing (P21) and install the contact retainer (P22). Install cable lugs (P23) on the open end and mount these to the PDB.
4. Install cable lugs (P23) to the BEC (P18) input wires and mount on the PDB.
5. Assemble a telemetry cable for connecting the winch controller to the autopilot using pre-crimped JST GH leads (P24) and JST GH receptacle housing (P25). Refer to design file *AWS-230-Winch.kicad_pro* and the autopilot documentation [34] for pin descriptions.
6. Assemble a telemetry cable for connecting the Herelink Air Unit (P5) with the autopilot using a suitable connector included with the autopilot. Refer to the autopilot and Herelink documentation for pinouts [34], [35].
7. Assemble a power cable for the Herelink Air Unit (P5) using the cable supplied with the unit and a servo cable (P26).
8. Connect cables to the autopilot using Table 7 as a reference. Route the wires using cable ties (P10) and *AWS-310-Cable_tie_mount* (P27) with double sided tape (P6). Care must be taken to ensure that no wire touches the autopilot as this can introduce vibrations to the controller. Fig. 3 can be used for reference.

**Table 7:** Autopilot connections. Cables that are integrated into the peripheral are marked with a “-“.

**Cube**	**Peripheral**	**Cable**
Port: POWER1	BEC	P28
Port: GPS1	Switch	Included with autopilot
Port: TELEM1	Herelink Air Unit UART	Instruction no. 6
Port: TELEM2	Winch controller	Instruction no. 5
Port: CAN2	Here3	–
Servo rail: RCIN	Herelink Air Unit S.bus	Included with Herelink Air Unit
Servo rail: Main out 1	Left front cyclic servo	–
Servo rail: Main out 2	Right front cyclic servo	–
Servo rail: Main out 3	Rear cyclic servo	–
Servo rail: Main out 4	Tail servo	–
Servo rail: Main out 7	Herelink Air Unit power	Instruction no. 7
Servo rail: Main out 8	ESC throttle input	Included with ESC
Servo rail: AUX out 4	ESC power out	Included with ESC
Servo rail: AUX out 6	ESC RPM out	Included with ESC

Assemble the battery boxes to complete the UAV build.

1. Cut the carbon fiber angle (P29) into 16 smaller brackets of 10 mm length.
2. Bond the brackets to *AWS-310-Battery_box_panel-Bottom* (P30), *AWS-310-Battery_box_panel* (P31) and *AWS-310-Battery_box_panel-Cutout* (P32) using the supplied fixture (P33-34) for ease of assembly. Use epoxy glue for the bonding (P35).
3. Bond *AWS-310-Battery_box_panel-Top* (P36) to the assembly using epoxy glue and the remaining 8 angle brackets. Use the supplied fixture (P34, P37) for ease of assembly.
4. Install *AWS-310-Battery_box_clip* (P38) to the top panel using M3x12 bolts and nuts.
5. Install *AWS-310-Battery_box_endplate-Cutout* and *AWS-310-Battery_box_endplate* (P39-40) on the bottom panel using tape and bond *AWS-310-Battery_box_clip_snag* (P41) to the front panel.
6. Cut foam (P42) sheets to cover the inside of each panel and apply spray adhesive (P43) to the inside of the battery box. Bond the foam to the box.
7. Insert a rubber band (P44) to *AWS-310-Battery_box_clip_snag* to close the two endplates.

Repeat the procedure above for the second battery box, skipping step 4.

### Winch

The first item during winch assembly is the catch assembly. The procedure is listed below.

**Fig. 12:**
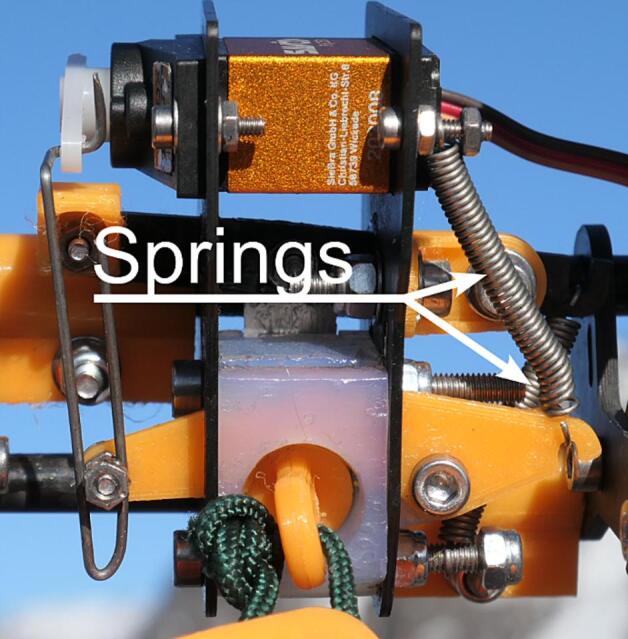
Springs installed on the catch assembly (bottom view).

1. Referencing the *AWS-200-Sensor_catch_assem* assembly file, cut *AWS-210-Blade* (P45) to a length of 15 using a grinder or Dremel. Take care to avoid cuts during the process. Glue the blade to *AWS-210-Blade_sheath* (P46) using epoxy glue (P35).
2. Proceed with assembly using the assembly file and the video *AWS-200-Sensor_catch_assem.mp4* as a reference.
3. Install a spring (P47) to both arms of the catch assembly; *AWS-210-Blade_arm* (P48) and *AWS-210-Sensor_catch_arm* (P49). See Fig. 12.

The pivot mechanism assembly is broken down into several steps. Each step is documented as small video files in the repository. Use the available assembly file for reference.

1. Cut the Ø6 mm carbon fiber tube (P50) into two pieces of 105 mm, each.
2. Start the pivot mechanism assembly by integrating the catch assembly as shown in the video file *AWS-200-Sensor_parking_assem_S1.mp4*, using the cut sections of carbon fiber tube.
3. Complete the guide ring assembly as shown in *AWS-200-Sensor_parking_assem_S2.mp4*. Note that *AWS-210-Guide_ring* (P51) is secured using double sided tape (P6).
4. Cut the Ø8 mm carbon fiber tubes (P52) into two pieces of 300 and 320 mm.
5. Complete the pivot mechanism assembly as shown in *AWS-200-Sensor_parking_assem_S3.mp4*.
6. Route the string included with the Reef 99 servo winch (P53) around the guide ring as show in the assembly file (string is marked in red). Tie the string to the Ø8 mm carbon fiber tube opposite the servo.

The winch unit subassembly follows next.

1. Disassemble the Penn Squall Low Profile reel (P54). Use the exploded view drawing included with the reel for reference. Parts 4, 6, 7, 10, 11, 13, 14, 15, 16, 17, 18, 101, 106, 107, 108 are used for the winch assembly.
2. Add threaded inserts (P55) to *AWS-Spool_bearing_block_left* and *AWS-210-Motor_mount_strut-30,3* (P56-57) using a hot-air gun or soldering iron to heat the insert prior to inserting.
3. The winch gears (P58-60) are modified stock spur gears. Modify the gears according to the included technical drawings (*AWS-210-Winch_gear-XXt.pdf*).
4. Solder the PCB (P61) according to the schematic and PCB layout file. Use the file *AWS-230-Winch.kicad_pro* as an entry point.
5. Assemble the winch unit as shown in the video files *AWS-200-Winch_Assem_S1.mp4* and *AWS-200-Winch_Assem_S2.mp4*.

The gimbal assembly is completed as outlined below.

**Fig. 13:**
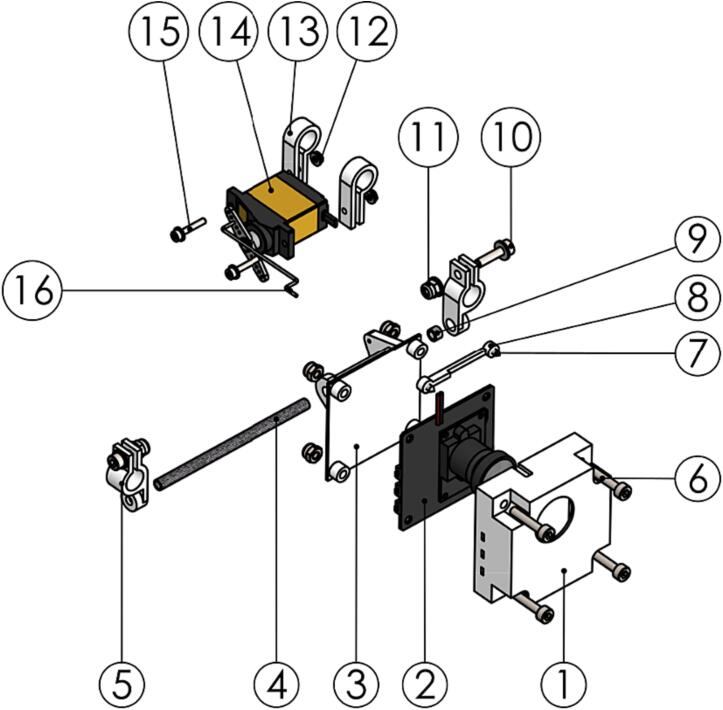
Gimbal assembly step 3 exploded view with (1) AWS-210-Hawkeye_housing, (2) HDMI camera, (3) AWS-210-Hawkeye_housing_lid, (4) Ø4 mm carbon fiber tube, (5) AWS-210-Gimbal_bracket, (6) M3x16 bolt, (7) 2.2x6.5 screw, (8) AWS-210-Hawkeye_sensor_PCB_clamp, (9) iglidur GSM-0304–03, (10) M3x12 bolt, (11) M3 locking nut, (12) M2 nut, (13) AWS-210-Gimbal_servo_bracket, (14) Savöx SH-0255MG, (15) M2x12 bolt and (16) AWS-210-Gimbal_rod marked.

1. Cut a 70 mm section from the Ø4 mm carbon fiber tube (P62).
2. Install the FFC jumper cable (P63) on the HDMI camera (P64) instead of the longer default cable.
3. Insert the camera into *AWS-210-Hawkeye_housing* (P65) and *AWS-210-Hawkeye_housing_lid* (P66) and attach it to the gimbal as shown in Fig. 13.

The two landing skid bow assemblies are identical and can be assembled according to the procedure below.

1. Assemble the bow as shown in Fig. 14. Take care to orient *AWS-210-Landing_skid_bow* (P67) correctly. The top recess should align with *AWS-210-Landing_skid_quick_release* (P68).

**Fig. 14:**
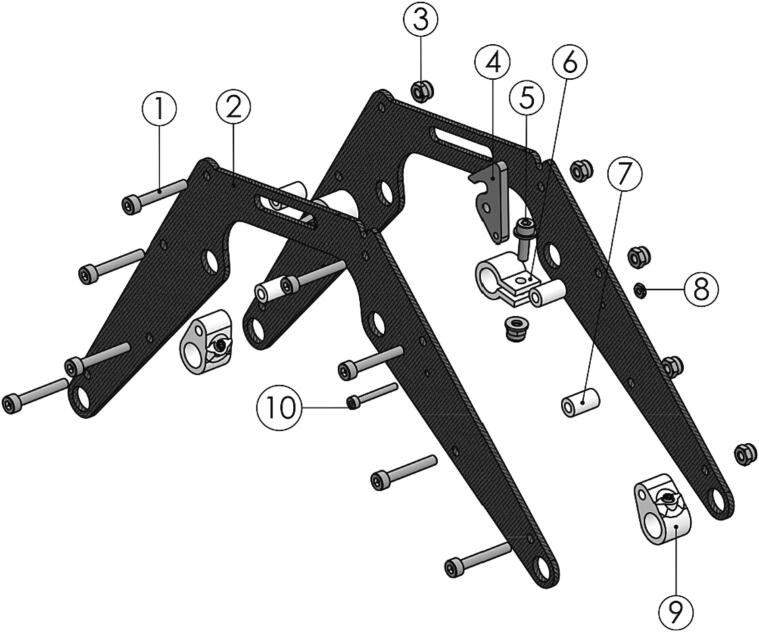
Landing skid bow exploded view step 1 with (1) M3x20 bolt, (2) AWS-210-Landing_skid_bow, (3) M3 locking nut, (4) AWS-210-Landing_skid_quick_release, (5) M3x10, (6) AWS-210-Tube_clamp-Sensor catch assem, (7) AWS-210-Landing_skid_brace, (8) M2 nut, (9) AWS-210-Landing_skid_T_joint and (10) M2x16 bolt marked.
2. Attach a spring (P47) between *AWS-210-Landing_skid_quick_release* and the M2 bolt (10).

The winch assembly can now be completed by joining the subassemblies as instructed.

1. Cut two sections of 300 mm of Ø8 mm carbon fiber tube (P52) to form *AWS-210-Landing_skid* (P69).
2. Combine the subassemblies build per the instructions above into the final winch assembly as shown in Fig. 15.

**Fig. 15:**
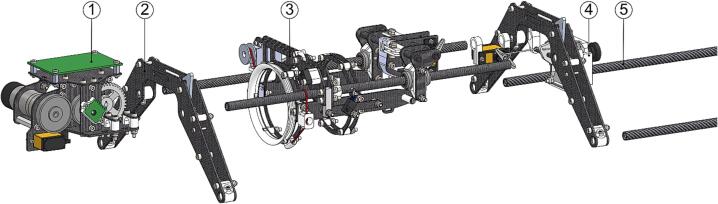
Winch assembly final build from subassemblies. (1) Winch unit, (2) skid bow assembly, (3) pivot and catch assembly, (4) gimbal assembly, and (5) AWS-210-Landing_skid.
3. Connect the PCB to external components using the instructions below. Refer to design file *AWS-230-Winch.kicad_pro* for pin descriptions.
  a. Solder a wire harness to the spool encoder PCB (P70) using pre-crimped JST GH leads (P24) and JST GH receptacle housing (P71). Connect to the PCB.
  b. Cut off the winch motor (P72) connector and solder pre-crimped Molex Nano-Fit lead (P19) to all wires. Insert the leads into a Molex Nano-Fit receptacle housing (P73) and install a retainer (P74) to secure the wires. Connect to the PCB.
  c. Solder pre-crimped Molex Nano-Fit lead (P19) to the camera power cables. Insert the leads into a Molex Nano-Fit receptacle housing (P21) and install a retainer (P22) to secure the wires. Connect to the PCB.
  d. Solder pre-crimped JST GH leads (P24) to the pivot limit switches (P75) and insert into JST GH receptacle housing (P76). Connect to the PCB.
  e. Solder a wire harness to the rangefinder (P77) using pre-crimped JST GH leads (P24) and JST GH receptacle housing (P71). Connect to the PCB.
  f. Connect the four servos (P53, P78) to the servo rail on the PCB. Use servo extension cables (P79) as needed.
4. Route and fasten wires securely, taking care to minimize any source of vibration or tension on cables during use.
5. Attach winch line (P80) to the winch spool using a trilene knot or another suitable knot. Route the line through the level wind, landing skid bow, *AWS-210-Wire_guide* (P81) and catch assembly. Tie a shackle (P82) to the end of the line.

Any payload that fits the required size and weight can be used. A SonTek CastAway CTD was used during testing, and assembling this specific payload is outlined below.

1. Fix AWS-510-CastAway_cage (P83) to the CTD (P84) using a M3x12 bolt and locking nut.
2. Tie the shackle (P82) to the CTD cage using a piece of paracord around the bolt.

The final assembly step can now be completed.

1. Install the batteries (P85) into the battery boxes and slide into the UAV battery bay.
2. Check the UAV center of gravity by attaching a digital pitch gauge (P86) to a horizontal part of the UAV (i.e. the motor top). Lift the UAV by the blade grips with the main blades perpendicular to the main frame.
3. Adjust battery position by sliding *AWS-310-Battery_box_clip* (P38) back and forth until the pitch gauge is within ± 0.2 degrees of level.
4. When battery adjustment is complete, install *AWS-310-Battery_endstop-Left* (P87) by drilling a hole in the lower right UAV frame and bolting the part in place, such that the endstop constrains the battery.

### Testing

Extensive testing of the UAV is needed to properly tune the autopilot for the specific aircraft. Use the ArduPilot documentation [33] for a complete guide of all steps involved. Failure to follow the instructions can lead to damage or injury.

The winch assembly can be tested extensively on the ground using the MAVLink Emulator program found in the repository. This enables testing the functionality of the winch while connected to a PC though USB with the UAV turned off, allowing for easier debugging of software and electronics.

## Operation instructions

A checklist, *AWS-910-Checklists.pdf*, has been included in the repository. This describes the use of the ARC-AWS through all stages of flight operation. It is recommended that the pilot of the UAV have significant experience flying other UAV systems before attempting to use the ARC-AWS. It is a complex and specialized tool that requires knowledge for proper and safe operation.

The checklist specifies handling the UAV while power is applied. Special care must be made as an accidental motor start can cause serious damage or injury. Familiarize yourself with the operation of the UAV without any blades installed before starting flight testing.

When taking off and landing, all personnel on the ground should be aware of the activity and stand clear of the landing site.

User input to the winch controller and UAV is though the buttons on the Herelink controller. Button mapping is described in Table 8.

**Table 8:** Herelink controller button mapping.

**Button**	**Short press**	**Long press**	**Channel**
A	Toggle: Slow reel out	Toggle: Dive command	Short: 6. Long: 7
B	Toggle: Slow reel in	Toggle: Fast reel in	Short: 9. Long: 10
C		Toggle: Cut command	11
D	Stabilize flight mode		MAVLink
Scroll wheel	Gimbal control		5
Camera button		Toggle: Motor Interlock	8

The CastAway CTD records the location when starting and stopping a measurement. As the CTD is started prior to takeoff (as specified in *AWS-910-Checklists.pdf*) the measurement location must be extracted from the UAV log recordings.

If the sensor payload is caught by ice the winch line can be cut by the pilot (see Table 8) to prevent damage to the UAV. This can also be used if the winch motor fails, and if it is not considered safe to fly with the sensor suspended below. The UAV can fly with an oscillating payload at lower speeds, but this has not been tested extensively.

The UAV batteries must be pre-heated when used in a cold environment to keep the internal resistance of the batteries low. Install batteries in the UAV immediately before flight to limit the temperature drop. During flight the self-heating during discharge and the battery box insulation keeps the battery at working temperature.

The UAV and winch assembly should regularly be serviced to ensure all moving parts are properly lubricated and/or show no sign of damage or wear. Failure to properly maintain the ARC-AWS can lead to serious damage or injury. Key items to check during regular service are listed below.

1. Main and tail rotor head bearings
2. Main head dampers
3. Main and tail blades
4. Main and tail rotor play
5. Gear wear
6. Wire integrity
7. Fastener loosening
8. Winch line wear
9. Pivot mechanism cord wear
10. Salt buildup from seawater
11. PCB connectors

## Validation and characterization

**Fig. 16:**
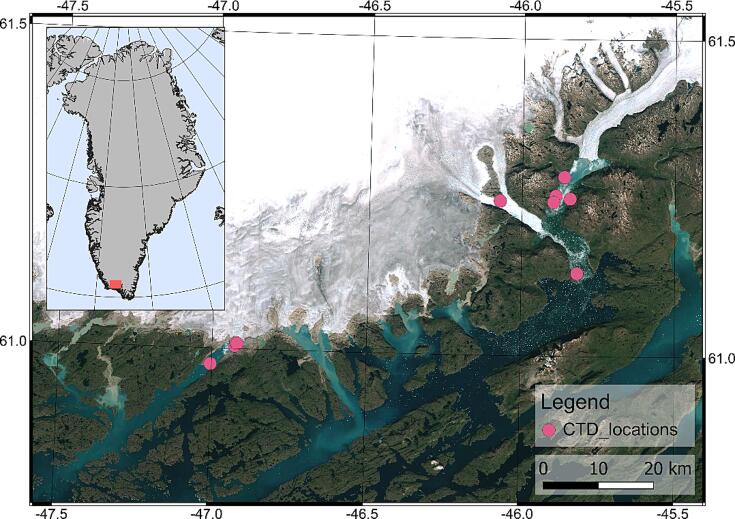
Locations of CTD profiles collected by the ARC-AWS.

The ARC-AWS was tested extensively in late March 2023 in South Greenland (see Fig. 16). CTD profiles were collected at glaciers Eqalorutsit Kangilliit Sermiat, Eqalorutsit Killiit Sermiat and Sermilik Bræ in conditions ranging from heavy ice mélange to open water. Transport to the operating locations was carried out in helicopters.

**Fig. 17:**
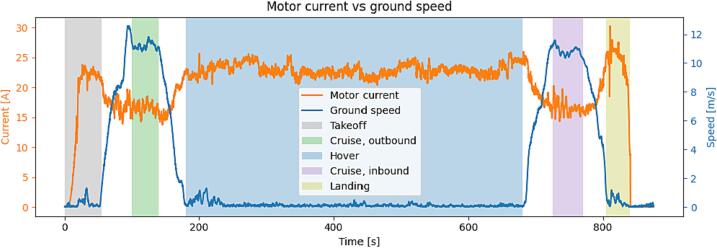
Comparison of UAV motor current and ground speed with the differen fligt segments highlighted.

Endurance testing was completed in the Narsarsuaq area, consisting of a complete flight in a stationary hover with fully charged batteries. When battery voltage reached 21.6 V the flight was stopped. The resulting usable flight time was found to be 24 min. The flight time for a mission profile with a large cruise portion will be significantly greater due to the decrease in power consumption in forward flight. This is obvious in Fig. 17 where the reduction in current consumption in cruise compared to hover is approximately 38 %.

**Fig. 18:**
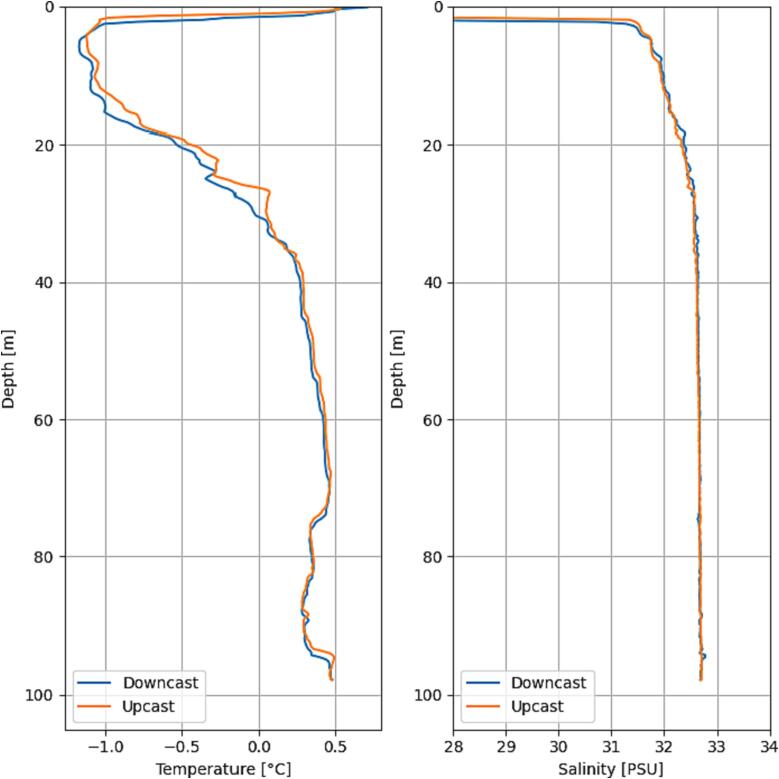
CTD data collected with the ARC-AWS during field work in South Greenland.

**Fig. 19:**
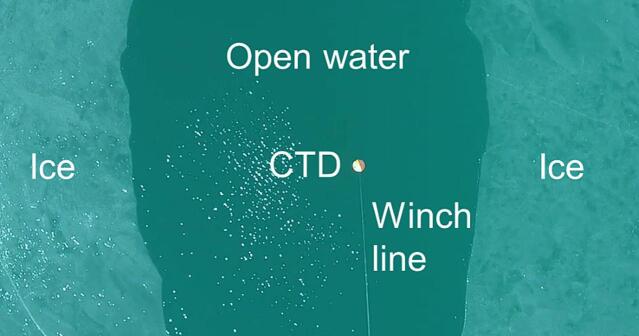
CTD just before profiling in a gap in ice mélange of approx. 2.2 m as seen from the UAV camera pointed straight down.

Fig. 18 shows data from one of the CTD profiles collected by the ARC-AWS during field work in South Greenland. The profile was taken in an area with significant ice mélange (see Fig. 19). Further, measurements were also collected approx. 5 m from Sermilik Bræ, highlighting the capabilities of the ARC-AWS.

Range testing of the radio control system was conducted at the coast south of Aarhus, Denmark with line-of-sight maintained during the test. Both controller and UAV was positioned between 3 and 6 m above sea level. The radio control and telemetry link was successful at a maximin distance of 11 km but a reliable video feed was only achieved at a maximum distance of 6 km.

### Performance assessment

During fieldwork the ARC-AWS was proven to be reliable, efficient, and easy to use for oceanographic profiling in remote and hazardous areas. To ensure the safest and most reliable mode of operation, two people are required for operation: a pilot and a spotter for maintaining visual contact. The system has demonstrated its capability to access areas not suitable for manned marine vessels, unlocking a potential for acquiring new and unique in-situ measurements of this hostile environment.

The ARC-AWS has been shown to be a great fit for missions requiring both stationary hover and extended cruise segments due to the high cruise efficiency. Missions primarily flown at cruise speeds could potentially allow flight times of up to about 35 min. Flights with large vertical segments (>100 m) were found to be most reliably flown at forward speeds above 4 m/s while descending and ascending. This allows for increased vertical speed as the rotor wake is trailing the UAV and translational lift is introduced thereby increasing efficiency and reducing instability. For laterally short missions this means extending the cruise beyond the measurement location. The autonomous winch was able to reliably reel the payload and allow the sensor to descent at terminal velocity.

### Improvements

Although successful, the ARC-AWS was found to be suitable for improvement in the following areas.

#### Payload and choice of aircraft

The current payload requirements are prohibitive for other possible use-cases for ARC-AWS. The pivot mechanism is not easily adapted to other payload sizes and shapes. Further, the winch unit and camera gimbal locations prohibit longer payloads. Repositioning the gimbal and winch unit is not easily done with the current aircraft as it is designed as a slim and tall frame with the battery incorporated within. Further, this does not easily allow expanding the battery capacity. A lower, wider frame could be better suited.

#### Gimbal

The camera and radio link used had connection issues when initially powering up the UAV. Numerous reboots solved the issue, but the time used allows the pre-heated batteries to cool, limiting flight time. Selecting a different camera that is better compatible with the radio equipment is needed to improve this. Further, the custom gimbal experience vibrations during flight due to flexibility in the gimbal structure. A commercial gimbal with integrated camera should be considered if a new iteration of ARC-AWS is to be made.

#### Weatherproofing

The current setup does not allow use in rain or snow conditions. The electronics of both UAV and winch are not protected in a weatherproof fuselage, mainly due to weight and development time constraints. This is an ideal area to improve to expand the usability of the ARC-AWS system. Further, incorporating a streamlined fuselage can potentially decrease the total system drag, allowing higher efficiency during cruise.

#### Increased RPM

The UAV rotor RPM was decreased to lower the power consumption of the UAV. This does, however, limit the available power for maneuvers and turbulence penetration. Furthermore, the tail authority is decreased as main and tail rotors are mechanically linked. This lack of power is obvious when i.e., rapidly stopping the UAV after high-speed cruise flight or when descending through the turbulent wake of the main rotor. The effect is increased mission time. If battery capacity is increased following the improvements above, the RPM can be increased as well, alleviating these issues.

#### Autopilot and wiring

The position of the autopilot has proven suited for easy access to wiring. However, the autopilot is not well protected during handling, potentially leading to damage. The UAV wiring is also not ideally secured as double-sided tape is used. Data and power cables from the UAV to the winch controller should be combined to ease deployment.

Further development and tuning of the autopilot can potentially allow operation from crewed marine vessels in moderate waves. This will unlock more possible use-cases.

## Ethics statements

Not relevant.

## CRediT authorship contribution statement

**Ebbe Poulsen:** Investigation, Methodology, Software, Validation, Visualization, Writing – original draft. **Søren Rysgaard:** Conceptualization, Methodology, Funding acquisition, Supervision, Writing – review & editing. **Karina Hansen:** Investigation, Project administration, Writing – review & editing. **Nanna B. Karlsson:** Conceptualization, Investigation, Funding acquisition, Writing – review & editing.

## Declaration of competing interest

The authors declare that they have no known competing financial interests or personal relationships that could have appeared to influence the work reported in this paper.
